# Brief early adolescent multi-family therapy (BEAM) trial for anorexia nervosa: a feasibility randomized controlled trial protocol

**DOI:** 10.1186/s40337-021-00426-4

**Published:** 2021-06-16

**Authors:** Julian Baudinet, Ivan Eisler, Mima Simic, Ulrike Schmidt

**Affiliations:** 1 Institute of Psychiatry, Psychology and Neuroscience (IoPPN), King’s College London, 16 De Crespigny Park, London, SE5 8AB UK; 2 Maudsley Centre for Child and Adolescent Eating Disorders, South London and Maudsley NHS Foundation Trust, De Crespigny Park, Denmark Hill, London, SE5 8AZ UK; 3 Adult Eating Disorders Service, South London and Maudsley NHS Foundation Trust, De Crespigny Park, Denmark Hill, London, SE5 8AZ UK

**Keywords:** Anorexia nervosa, Adolescents, Multi-family therapy (MFT), Family therapy, Family based treatment (FBT), Maudsley family therapy, Randomized controlled trial, Feasibility, Group, Protocol

## Abstract

**Introduction:**

Multi-family therapy (MFT) is a recommended treatment for adolescent anorexia nervosa internationally. Despite recent significant advances in single-family therapy, the evidence base for MFT remains relatively small. Several individual and family factors have been associated with poorer outcomes in single-family therapy, many of which may be addressed or ameliorated by MFT if delivered early in treatment. This trial aims to determine the feasibility and acceptability of adding a five-day multi-family therapy group to the early stages of family therapy for anorexia nervosa. Secondary objectives are to explore effect size changes in key individual and family factors across treatment.

**Methods:**

This feasibility trial will use a randomised controlled design. Sixty adolescents (age 10–17 inclusive) with anorexia nervosa or atypical anorexia nervosa and their parents will be recruited from a community-based specialist eating disorder service in London, UK. Participants will be randomly allocated to receive six months of eating disorder focussed family therapy with a five-day MFT group (experimental group) or without (control group). Block randomisation will be conducted by the King’s Clinical Trials Unit and researchers will be blind to participants’ intervention allocation. Feasibility, acceptability and secondary outcomes measures will be collected at baseline, post-MFT, end of treatment, six-month and 12-month follow-up. Feasibility and acceptability will be assessed according to trial sign-up rates, retention, measure completion rates and satisfaction. Secondary outcomes include physical health improvements, changes in psychiatric symptoms, emotion regulation and reflective function capacity, expressed emotion, parental difficulties and therapeutic alliance. Descriptive data and exploration analysis of trends and effect sizes will be reported upon at trial completion.

**Discussion:**

The five-day MFT program developed for this study is novel, brief and more accessible than previous MFT models. The inclusion of a data collection point during treatment and follow-up will allow for an investigation of trends during and after treatment. This will allow exploration and comparison of future potential mediators and moderators of MFT and FT-AN outcomes and how these may differ between treatments.

**Trial registration:**

ISRCTN registry; ISRCTN93437752, on 27 January 2021.

**Supplementary Information:**

The online version contains supplementary material available at 10.1186/s40337-021-00426-4.

## Background

Anorexia nervosa and atypical anorexia nervosa are serious psychiatric disorders that have a significant impact on the individual and family. They are associated with high levels of distress, personal and social impairment and reduced quality of life [1]. Adolescence and early adulthood is when eating disorders typically develop and early detection is key to improving outcomes and treatment efficacy [2, 3]. Family therapy focused on anorexia nervosa (FT-AN) is the first line recommended treatment for adolescents internationally [4] and has been shown to be superior to individual approaches [5]. Nevertheless, a significant minority of young people do not respond to current treatments [6, 7] and go on to develop a more chronic course of the illness associated with high rates of disability and mortality [8].

There is emerging evidence that a range of factors are associated with treatment outcomes [9]. One consistent finding is that the early stages of treatment appear very important. Weight gain within the first few weeks of treatment has now consistently been shown to predict improved outcomes at the end of treatment [10–13]. This fits with findings that six months of family treatment is as effective as 12 months [14]. Family factors, such as increased levels of expressed emotion (criticism, hostility, emotional overinvolvement) [15] and low parental self-efficacy [16, 17] have also been identified as potentially reducing treatment response. Individual factors, including eating disorder symptom strength and level of obsessionality [17] may moderate outcome. Finally, therapeutic alliance may also mediate outcomes [18–20]. Nevertheless, these findings have not always been replicated and studies are often under powered [5, 9], making it difficult to confidently identify the specific impact these factors may have. More broadly within the eating disorder field, comorbidity has also been shown to predict poor treatment outcomes [21] and early behaviour change predicts later symptom remission [22], highlighting the potential importance of addressing these factors within adolescent treatments.

Intensive versions of FT-AN have recently been developed in an attempt to target some of these factors, reduce treatment length and improve outcomes. Different centres have explored the impact of intensive single- [23, 24], as well as multi-family therapies (MFT) [25–27], both of which are based in the principles of FT-AN. MFT is a group-based treatment for up to eight families simultaneously in one group [28, 29]. The group works together with the support of a therapeutic team over the course of eight to 10 days spread across six to nine months [27, 30]. It is designed to improve treatment outcomes by reducing isolation, promoting solidarity and increasing treatment intensity [27, 31], all of which are uniquely afforded by the multi-family setting and may help to improve outcomes above and beyond what can be expected in the single-family format. It has recently been manualised for adolescents [32] and is a recommended treatment for adolescent anorexia nervosa internationally [33–35]. MFT has also been developed for young adults with anorexia nervosa [36] and adolescents with bulimia nervosa [37].

There is now emerging evidence that MFT for adolescent anorexia nervosa is associated with a reduction in eating disorder symptoms [38–41], improved mood and self-esteem [42], quality of life [39], family functioning [43], reduced carer burden [44], expressed emotion [45], and improved motivation and familial communication [46]. The addition of MFT to FT-AN also leads to improved outcomes [38], indicating the manualised 10-day MFT model may be superior to current first-line treatments in promoting and maintaining weight gain. There is also evidence that a stand-alone week-long intensive MFT on its own may lead to symptom improvements [26, 47].

Despite these promising emerging findings, the evidence base for MFT is small. There is a noticeable dearth in controlled trials, with only one published randomised controlled trial (RCT) (*N* = 169) in an outpatient setting [38], one small inpatient RCT (*n* = 25) [48], and one published RCT protocol [49]. There is also large variability between studies in the way MFT is delivered, including; setting (inpatient, day programme, outpatient), how many days are offered, the frequency of MFT days and the duration of treatment [40]. Shorter courses of MFT have been offered in some studies [26, 47] due to feedback from service users, the resources required and the cost of offering 10 full days of treatment. Furthermore, there have been no studies investigating potential treatment mechanisms, predictors, moderators or mediators of MFT treatment outcome to date.

Given MFT offers the opportunity for early, family focused, intensification of treatment, it is well placed to target and address several of the identified moderators and mediators of FT-AN outcomes. It also has the potential to target these at the most crucial early stages of treatment to promote early change. Recent evidence suggests that five days of MFT may be sufficient, although the impact on outcomes at end of treatment is unknown. The current trial aims to determine whether adding a five-day intensive version of MFT is feasible within the early stages of FT-AN and to explore effect size changes of potential key outcome, moderator and mediator variables. This will allow for the development of briefer, and potentially more efficient, intensive treatment options that are less resource intensive.

### Study aims

#### Primary objective

The primary objective of this study is to examine the feasibility and acceptability of adding a five-day MFT intensive week within the first two months of FT-AN using a randomised controlled design. It is hypothesised that five-day MFT will be both feasible and acceptable to adolescents and their families.

#### Secondary objectives

The secondary aims are to examine the effect size changes of potential moderators and mediators associated with poorer outpatient treatment response (eating disorder and comorbid psychiatric symptoms, general and family functioning, therapeutic alliance/engagement, parental difficulties, and mentalising and emotion regulation capacity) to inform a later full-size RCT. Hypotheses were not generated for secondary objectives due to their exploratory nature.

## Methods and analysis

Protocol version 7, 04.02.2021.

### Design

**Fig. 1:**
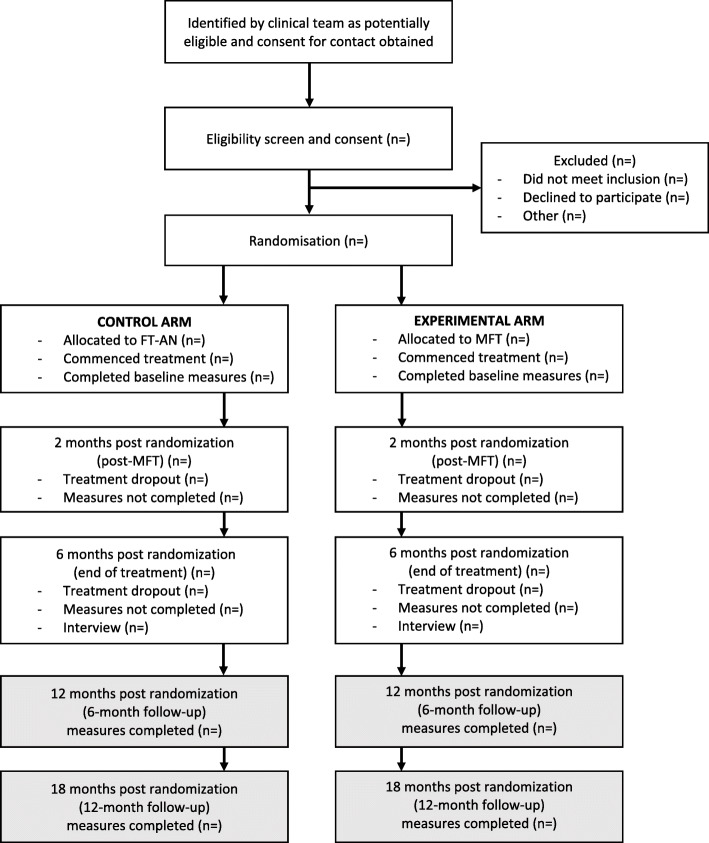
Participant Flowchart

A single-site feasibility randomised controlled trial (fRCT) design will be used. The control group will receive six months of FT-AN. The experimental group will receive six months of FT-AN plus a five-day intensive MFT delivered within the first two months of treatment. See Fig. 1 for participant flowchart. The Standard Protocol Items: Recommendations for Interventional Trials (SPIRIT) checklist [50] was used in the design of this study. See supplementary material for completed checklist.

### Setting

This trial will be conducted within a single specialist community-based child and adolescent eating disorder service in London, UK.

### Patient and public involvement (PPI)

Design of the MFT intervention was based on feedback from several initial pilots of intensive MFT. Patients and carers satisfaction was good and both reported finding the brief form of MFT helpful. The trial methodology was then designed in consultation with the clinical service’s research steering group, which has active ongoing PPI representation.

### Participants

Inclusion criteria:

I. Aged 10–17 years, inclusive
II. DSM-V diagnosis of anorexia nervosa or atypical anorexia nervosa with rapid weight loss (< 15%mBMI over three months)
III. Medically and psychiatrically fit for outpatient treatment

Exclusion criteria

I. Active psychotic illness
II. No available parents/guardians to participate in treatment
III. Safeguarding concerns that make conjoint family therapy contraindicated

### Recruitment

The study aims to recruit 60 young people and their families (30 per arm). This is based on guidance from the National Institute for Health Research that a sample size required for a fRCT ranges from 24 to 50 participants [51–54] and other guidance that a sample size as large as 70 may be required [55]. The recruitment site is a research active service. To achieve adequate enrolment in the trial a member of the research team will regularly attend team meetings to answer any questions about the study and identify potential participants.

### Consent

Families who consent to be contacted by the research team will be provided the Participant Information Sheet (PIS). They will have the study clearly explained to them and any questions answered. Once all questions have been thoroughly answered the participant’s eligibility is screened based on the inclusion criteria. If eligible, participants complete the consent form to join the trial. Baseline assessment instruments are then provided. This process can occur in person or via secure video-link. Parents and adolescents over 16-years-of-age consent individually. Children under age 16 sign an assent form alongside parental consent for them to participate. Developmentally appropriate material has been developed for this age group.

### Randomization

Block randomisation will be performed by the Clinical Trials Unit at King’s College London who are not involved in any aspect of data collection or analysis for this study. No demographic or baseline characteristics will inform the randomisation process. Computer generated block randomisation, with random block sizes, will be used to ensure both arms of the fRCT are equal. Treatment allocation will be securely, electronically, delivered to one, un-blinded, member of the research team who will communicate this with the clinical team. This person will not be involved in any aspect of data collection or analysis.

### Blinding and concealment

The researchers, data managers, and statisticians will remain blind to each participant’s experimental group allocation. Treatment allocation will be provided directly to the clinical team. Blinding of the participants and treatment team will not be possible as they will be aware of the treatment they are receiving by participating in it.

### Intervention

All participants (in both study arms) will receive FT-AN [56] the current first-line recommended treatment [33]. This means all participants will receive the best available care. The experimental group will additionally receive a modified version of MFT [32], another recommended treatment by the National Institute for Health and Care Excellence (NICE) [33]. Both treatments have been shown to be safe and effective for this group [5] although the feasibility and efficacy of the modified version of MFT proposed in this study have not.

#### Family therapy for anorexia nervosa (FT-AN)

FT-AN is a specific, manualised, evidence-based, four phase treatment for adolescent anorexia nervosa [56]. Young people and their families are initially seen weekly, which becomes less frequent as treatment progresses. Treatment initially focuses on engagement and supporting the family to manage the eating disorder symptoms. Once the young person is managing food more effectively and is more stable medically, treatment shifts to developmental and family lifecycle needs. FT-AN is delivered by an eating disorder clinician trained in the treatment model over six months.

#### Five-day intensive multi-family therapy (MFT)

The brief, five-day intensive version of MFT used in this fRCT is an adaptation of the manualised 10-day treatment [32]. During MFT, up to eight families working together with a clinical team over the course of a week to build skills, promote engagement and increase understanding around the illness and family dynamics. The brief intensive version also includes between two and eight families per group and condenses the main treatment content into five full consecutive days (10 am-4 pm) over one week (Monday to Friday). It is delivered by two clinicians. Treatment content matches the phases of FT-AN and focuses on empowering parents to support their child to manage eating disorder symptoms and restore weight. See supplementary material for treatment details, including an example timetable of the five-days.

#### Training and supervision

All clinicians working on this trial will receive the relevant training in FT-AN and/or MFT-AN. Regular model-specific supervision will be provided by the developers of both treatments throughout the trial period. This will help improve adherence to treatment protocols, improving treatment fidelity.

#### Concomitant treatments and post-trial care

Concomitant psychological treatments are not permitted during this trial. Psychopharmacological treatment is permitted. Medication type, dose and duration will be recorded and reported on. Additional, adjunctive treatments will be available within the same specialist service if any participant should require additional treatment at the end of the six-month treatment trial period. Data on type, duration and intensity of additional treatments will be collected and reported on as part of follow-up data.

### Outcome reporting

**Fig. 2:**
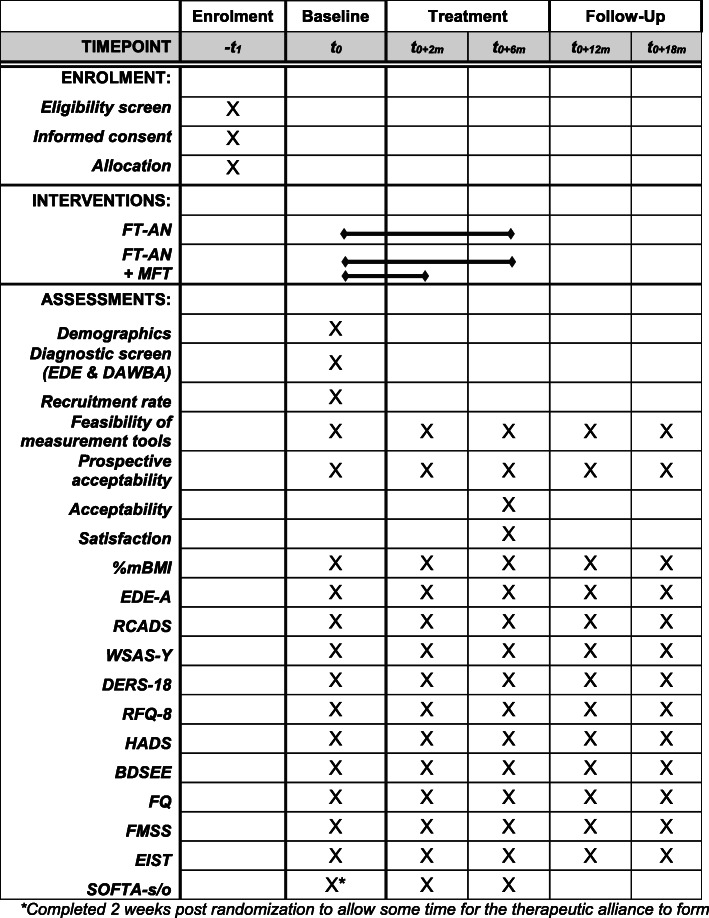
Schedule of enrolment, interventions, and assessments (SPIRIT figure). *Completed two weeks post randomization to allow some time for the therapeutic alliance to form

All participants will complete a battery of self-report and observational assessments at five timepoints; baseline, post-MFT (two months post randomisation), end of treatment (six months post randomization), six-month follow-up (12 months post randomization) and 12-month follow-up (18 months post randomization). See Fig. 2 for details.

#### Baseline diagnostic assessment

Participants will complete the Eating Disorder Examination [57] diagnostic interview to confirm current eating disorder diagnosis. Participants will also complete the Development and Wellbeing Assessment [58] online diagnostic screens to confirm current comorbid Diagnostic and Statistical Manual [59] psychiatric diagnoses. Both are widely used, validated diagnostic instruments used with children and adolescents.

#### Primary outcome

Feasibility and acceptability will be assessed using five domains. See Table 1 for each domain and how they will be reported on.

**Table 1:** Feasibility and acceptability domains and outcome reporting

*Domain*	*Metric*
Recruitment rate	- Percentage of eligible participants who consent to participate in the study during the study period (18 months)
Feasibility of measurement tools	- The mean time taken to complete questionnaires in minutes. - Amount of missing data per questionnaires reported as a percentage per measure - The number of participants who complete questionnaires at two months, end of treatment (six months), six-month follow-up and 12-month follow-up reported as a percentage of the total sample
Prospective acceptability	- Free text responses for why individuals did not take part or discontinued the trial - Mean responses to the Expectation of Improvement and Suitability of Treatment (EIST). The EIST is a two-item measure assessing how successful and how suitable the participant thinks treatment is.
Acceptability of intervention for participants	- The total number of sessions attended recorded at the end of treatment (six months) - The total number of sessions cancelled with reason recorded at the end of treatment (six months) - The total number of sessions participants did not attend without provided notice recorded at the end of treatment (six months)
Participant satisfaction	- Interview responses during qualitative interviews at the end of treatment (six months).

#### Secondary outcomes

To explore secondary outcomes, effect size changes will be explored across treatment and follow-up for eating disorder and comorbid symptomatology, general functioning, reflective functioning, parental coping, emotion regulation, expressed emotion and therapeutic alliance. All instruments will be completed at all five time points, except for therapeutic alliance which is not assessed at follow-up timepoints**.**

##### Anthropometric assessment

Height will be measured at beginning and end of treatment by trial researchers. Weight is measured weekly in FT-AN as well as on the first and last day of MFT by clinical staff. Weight and height will be used to calculate percentage of median Body Mass Index (%mBMI) adjusting for age and sex.

##### Psychological symptoms

The Eating Disorder Examination Questionnaire for Adolescents (EDE-A) [60] is a validated instrument of eating disorder symptoms with available adolescent norms [60]. The Revised Children’s Anxiety and Depression Scale (RCADS) [61] will be completed by adolescents and parents to assess depression and anxiety symptoms. The RCADS has been used extensively and shown to have good psychometric properties [62].

##### General functioning

Both parents and adolescents rate the adolescent’s general functioning using the Work and Social Adjustment Scale for Youth (WSAS-Y) [63]. This five-item scale assesses five domains of general functioning: school, daily skills, social, hobbies and family. It has been shown to have good internal consistency and test-retest reliability [63].

##### Reflective functioning

The Reflective Function Questionnaire (RFQ) [64] will be included to measure adolescent’s and parents’ own mentalization and reflective function capacity. It has been shown to be a valid measure of mentalizing capacity [64].

##### Emotion regulation

Adolescent and parents’ own emotion regulation capacity will be assessed using the 18-item Difficulties in Emotion Regulation Scale (DERS) [65]. This is a widely used, validated measure of emotion regulation capacity in adolescents and adults [66, 67]. It has been used recently with an adolescent eating disorder population [20].

##### Parental mood and anxiety symptoms

The Hospital Anxiety and Depression Scale (HADS) [68] is a validated and reliable measure of adult anxiety and depression symptoms in the community setting [69]. It has shown good factor structure and internal consistency [70].

##### Expressed emotion

Level of expressed emotion will be measured using three instruments; one adolescent and two parent-focused. Adolescents will complete the Brief Dyadic Scale of Expressed Emotion (BDSEE) [71], which includes three subscales; criticism, emotional overinvolvement and warmth. The BDSEE has been shown to have good reliability and validity in adolescent and eating disorder populations [71, 72]. Parent’s own perceived level of criticism and overinvolvement will be assessed using the self-report Family Questionnaire (FQ) [73], a valid measure of expressed emotion [73] Parents also complete the verbal Five Minute Speech Sample (FMSS) [74] task. This requires each parent/carer to talk about their thoughts and feelings about the patient for five uninterrupted minutes. This speech is recorded and later coded for overall level of expressed emotion. This is considered a valid measure of expressed emotion [75]. The FMSS will be used to identify EE categories (high/low criticism, high/low emotional overinvolvement, positive/negative relationship). Level of criticism and emotional overinvolvement (continuous variables) will be derived from the BDSEE and FQ for young people and parents, respectively.

##### Therapeutic Alliance

The System for Observing Family Therapy Alliances (SOFTA) [76] will be used to measure therapeutic alliance with the young person as well as parents. The SOFTA includes both a 16-item self-report instrument (SOFTA-s) and an observational tool (SOFTA-o) to analyse both audio and video recordings [77]. Both have been approved for use in this study. The self-report instrument will be used primarily and the observational tool only if data are missing. The SOFTA is widely used and validated for use in family therapy [76]. Baseline SOFTA assessment is completed at two-weeks post randomization, not initial assessment, given therapeutic alliance requires some time in treatment to develop. It is not completed at either six- or 12-month timepoints.

### Data collection, management and confidentiality

All participants will be assigned a unique trial identification number used for randomisation, data collection and analyses. All anonymised data will be stored securely and separately to consent forms in password-protected computers only accessible to the research team. If participants discontinue, weight and treatment characteristics will continue to be collected if consent is provided. Participants are provided with a small financial incentive (£10 voucher) if they complete measures at all five assessment time points.

### Analysis plan

Primary outcomes (feasibility and acceptability) will be reported using means, standard deviations and ranges, or medians and interquartile ranges of the primary outcomes listed above (see Table 1). Numeric feasibility and acceptability parameters (recruitment rate, missing data, loss to follow-up, treatment dropout) will be categorised as either green (> 75% response rate), amber (50–75% response rate), or red (< 50% response rate). Numeric values will also be considered in the context of non-numeric/free-response data where available. Parameters in the red and amber zone will be carefully considered and/or amended for the design of the main trial.

Linear mixed models [78] will be used to explore any changes in secondary outcomes (young person and parent factors) over the treatment and follow-up period. These models are commonly used statistical methods to analyse longitudinal data [79]. Simulation studies have shown them to be robust with moderate sample sizes, as in this study, when the data are normally distributed or lightly skewed [80–82]. This approach allows for random factors (both between and within-subject variability) to be included, meaning individual differences at baseline (e.g., demographic and illness characteristics) can be taken into account, as well as the possibility of individual differences in change trajectories during treatment.

Change in weight will also be calculated for the subgroup who are underweight at baseline (< 90%mBMI) or for whom weight gain is an identified treatment target. Baseline characteristics (gender, illness severity and duration, prior treatment, intact family status) and post-treatment characteristics (additional treatment type and dose, re-referral to services, referral to higher levels of care) will also be reported and groups compared using exploratory t-tests. Group differences between those with available data and those with missing data will also be reported and compared using exploratory t-tests. Together, this will be used to identify variables to be considered as strata for randomisation in the larger trial.

### Oversight and monitoring

Trial-related monitoring, audit and reviews may be conducted by the sponsor and research ethics committee at any time, who may request and access source data and other documents. The sponsor or chief investigator may prematurely discontinue the trial at any time. No adverse events are anticipated given the widespread use of both FT-AN and MFT internationally. If indicated, additional treatment can be offered once participants have completed the trial. Trial updates will be regularly provided to the service research steering committee. A data monitoring committee has not been established due to the small size of this trial.

## Discussion

This trial will determine whether it is feasible and acceptable to add a five-day multi-family therapy group to the critical early stages of family therapy for anorexia nervosa. This will inform the design of a full-size RCT to test whether the addition of brief-early MFT to FT-AN improves treatment outcomes for adolescents with anorexia nervosa.

This trial has several strengths. The use of a randomised controlled design will add rigour to the existing MFT evidence base as only one outpatient controlled trial has currently been reported on to date [38]. Additionally, it is the first MFT study to include data collection points during as well as at the end of treatment. This will help explore potential patterns of change during MFT and FT-AN treatment. Similarly, the inclusion of follow-up data collection points also has the potential to help advance the field by increasing our understanding of the process of change during and after both treatments. Lastly, the trial design allows for exploration of effect size changes of some of the key mediators and moderators of treatment outcome [9], which will extend future understanding of potential MFT treatment mechanisms. This will ensure the full-size RCT will be better equipped to be assessing the most suitable variables and in the most acceptable and feasible manner, with the greatest likelihood of being as effective as possible.

Another strength is that the current trial is investigating and testing more efficient, briefer MFT treatment. The five-day MFT model is less resource intensive and likely more manageable for services to offer than the current manualised 10-day model. If it is assessed as feasible and can be rigorously tested in a larger RCT it has the potential to be more broadly integrated within services and may emerge as a new, accessible intensive treatment option.

Despite these strengths all conclusions drawn from this trial will be tentative given the small sample size and number of measures included. Another limitation is that there is only one recruitment site, meaning site specific factors cannot be determined. Another potential limitation is that MFT is added within the first two months of treatment, rather than the first month. Increasingly, data indicates early change within the first month specifically impacts end of treatment outcomes [11, 12], suggesting intensive MFT may be more effective the earlier it is delivered. Due to service constraints and referral rates, having MFT within the first month is unfeasible.

### Trial status

The trial was registered on ISRCTN registry; ISRCTN93437752, on 27 January 2021. Ethics approval was granted for this project in August 2020 by the Stanmore Research Ethics Committee London (IRAS: 234354; REC: 20/LO/0839). Trial recruitment is currently suspended due to the novel coronavirus (COVID-19) pandemic. Recruitment will commence once group-based work is approved by local authorities.

## Supplementary Information

**Additional file 1.**

**Additional file 2 Supplementary Table 1** | Example five-day intensive multi-family therapy activity timetable*.

## Data Availability

Data will be made available upon request for those participants who have consented to this.

## Abbreviations

%mBMI: Percentage of median body mass index
BDSEE: Brief dyadic scale of expressed emotion
BEAM: Brief, early adolescent multi-family
COVID-19: Novel coronavirus 2019
DERS: Difficulties in emotion regulation scale
EIST: Expectation of improvement and suitability of treatment
FMSS: Five minute speech sample
FQ: Family questionnaire
fRCT: Feasibility randomized controlled trial
FT-AN: Family therapy for anorexia nervosa
HADS: Hospital anxiety and depression scale
IRAS: Integrated research application system
ISRCTN: International Standard Randomised Controlled Trial Number
MFT: Multi-family therapy
NICE: National Institute for Health and Care Excellence, UK
PIS: Participant information statement
PPI: Patient and public involvement
RCADS: Revised children’s anxiety and depression scale
RCT: Randomized controlled trial
RFQ: Reflective function questionnaire
SOFTA: System for observing family therapy alliances
SOFTA-o: System for observing family therapy alliances observational tool
SOFTA-s: System for observing family therapy alliances self report tool
SPIRIT: The standard protocol items: recommendations for interventional trials
WSAS-Y: Work and social adjustment scale for youth
