# Supplementary material for: Brief early adolescent multi-family therapy (BEAM) trial for anorexia nervosa: a feasibility randomized controlled trial protocol
Source: J Eat Disord. 2021 Jun 16;9:71. doi: 10.1186/s40337-021-00426-4 (PMC8206871; doi:10.1186/s40337-021-00426-4)
Supplement: Supplementary file 2 — Additional file 2 Supplementary Table 1 | Example five-day intensive multi-family therapy activity timetable*. [file 40337_2021_426_MOESM2_ESM.docx]

**Supplementary Table 1** | Example five-day intensive multi-family therapy activity timetable*

|  | **Day 1** | **Day 2** | **Day 3** | **Day 4** | **Day 5** |
| --- | --- | --- | --- | --- | --- |
| **Time \ Theme** | **Engagement & Education** | **Meals & Symptom Mgmt.** | **Thoughts & Emotions** | **Relationships (& how to manage as a family)** | **Looking ahead / contracting** |
| **09.30-10.00** | **weight** |  |  |  | **weight** |
| **10.00-10.15**  (ice Breaker) | YP to introduce family and talk about favourite thing to do together (after small huddle) | Reflections from yesterday | Non-Dominant Hand task | Welcome siblings  Family headlines | No ice breaker. Straight into activity |
| **10.15-11.00**  (main activity) | Picture card task | Sunday lunch | Brain scans | Family sculpt | Timelines |
| **11.00-11.30** | Snack | Snack | Snack | Snack | Snack |
| **11.30-13.00** | *SPLIT GROUP*  YP: portraits of AN; pros and cons  Parents: Lunch planning | Mealtime role play | Feedback from brain scans | *SPLIT GROUP*  YP: Motivation see saws and making traps and treasures Parents: Where do we stand? | Feedback on timelines (incl. contracting for future treatment) |
| **LUNCH** | Lunch | Foster family lunch | Lunch | Lunch | Lunch |
| **BREAK** | **Have games / distractions available for families** | | | | |
| **14.00-15.00** | Feedback from pros and cons (in stations) | Feedback from foster families (20-30m)  *SPLIT GROUP* Mums/dads/YP/Siblings: Create top 10 list of things to make meal times work from their perspective | *SPLIT GROUP (30-40m)*  YP: Care tags  Parents: Validation  Feedback from care tags (parents practice validation) | Family crest  *OR*  Speed problem solving | *SPLIT GROUP*  Toolboxes for the future |
| **15.00-15.30** | Snack | Snack | Snack | Snack | Snack |
| **15.30-16.00** | Relaxation task; mindfulness | 3 balls mindfulness - link debrief to post-meal activities and why they are important (and link to neurobiology) | Pebble & Balloon | Traps and treasures | Picture card task (reflections) |

*Abbreviations: YP; young person*

**See Simic, et al. (2021) for details of each activity*
